# Mean Normalized Gain: A New Method for the Assessment of the Aerobic System Temporal Dynamics during Randomly Varying Exercise in Humans

**DOI:** 10.3389/fphys.2017.00504

**Published:** 2017-07-18

**Authors:** Thomas Beltrame, Richard L. Hughson

**Affiliations:** ^1^Department of Kinesiology, University of Waterloo Waterloo, ON, Canada; ^2^Conselho Nacional de Desenvolvimento Científico e Tecnológico (CNPq) Brasília, Brazil; ^3^Department of Physiotherapy, Universidade Ibirapuera São Paulo, Brazil; ^4^Schlegel-University of Waterloo Research Institute for Aging Waterloo, ON, Canada

**Keywords:** oxygen uptake kinetics, frequency domain, PRBS, aerobic system, fitness

## Abstract

The temporal dynamics of the oxygen uptake ($\dot{V}O_{2}$) during moderate exercise has classically been related to physical fitness and a slower $\dot{V}O_{2}$ dynamics was associated with deterioration of physical health. However, methods that better characterize the aerobic system temporal dynamics remain challenging. The purpose of this study was to develop a new method (named mean normalized gain, *MNG*) to systematically characterize the $\dot{V}O_{2}$ temporal dynamics. Eight healthy, young adults (28 ± 6 years old, 175 ± 7 cm and 79 ± 13 kg) performed multiple pseudorandom binary sequence cycling protocols on different days and time of the day. The *MNG* was calculated as the normalized amplitude of the $\dot{V}O_{2}$ signal in frequency-domain. The *MNG* was validated considering the time constant τ obtained from time-domain analysis as reference. The intra-subject consistency of the *MNG* was checked by testing the same participant on different days and times of the day. The *MNG* and τ were strongly negatively correlated (*r* = −0.86 and *p* = 0.005). The *MNG* measured on different days and periods of the day was similar between conditions. Calculations for the *MNG* have inherent filtering characteristics enhancing reliability for the evaluation of the aerobic system temporal dynamics. In conclusion, the present study successfully validated the use of the *MNG* for aerobic system analysis and as a potential complementary tool to assess changes in physical fitness.

## Introduction

The study of the oxygen uptake ($\dot{V}O_{2}$) kinetics deals with the ability of data modeling to describe, in mathematical terms, the temporal characteristics of the aerobic response to the challenge of a step increase in work rate (*Ẇ*) (Hughson, 2009). Time-domain kinetic analysis has some limitations due to the white Gaussian noise associated with breath-by-breath fluctuation (Lamarra et al., 1987) that adds uncertainty to time-domain index predictions estimated from a single test dataset. To increase signal-to-noise ratio, studies commonly repeat similar tests multiple times within the same session (Ozyener et al., 2001; Beltrame et al., 2016; Christensen et al., 2016) or on different days (Whipp et al., 1982; Keir et al., 2014a) and average repetition-like transitions before time-domain data modeling. However, it is time consuming to repeat multiple similar exercise protocols until reproducible data are obtained. In addition to multiple repetition-like transitions, studies have applied frequency-domain data filtering before time-domain kinetic analysis (Harper et al., 2006; Schlup et al., 2015). Despite reducing the confidence interval of estimated time-domain parameters, the exponential data fitting procedure after filtering still deals with explicit modeling where model parameters are assumed *a priori* (Eßfeld et al., 1987). Therefore, new methods for the extraction of indexes related to aerobic system temporal dynamics without the need of model assumption should be investigated.

An attractive alternative to multiple repetitions of step transitions for evaluating the kinetic behavior of the aerobic energy supply system is the pseudo-random binary sequence (PRBS) in which *Ẇ* varies between two levels which are normally constrained to the light to moderate intensity exercise domains (Eßfeld et al., 1987; Hughson et al., 1990; Beltrame and Hughson, 2017a). The $\dot{V}O_{2}$ response to PRBS protocols is evaluated in the frequency-domain filtering out non-periodic signals associated with white noise, improving the extraction of parameters related to the aerobic system dynamics. The influence of inter-breath noise in the $\dot{V}O_{2}$ dynamics commonly occurs at higher frequencies that can be neglected during the frequency-domain analysis, theoretically increasing the biological significance of the estimated indexes. The attractiveness of the PRBS approach results from the potential to gain a quantitative index of kinetics from fewer exercise testing sessions in comparison to time-domain approaches (Hughson et al., 1991; Yoshida et al., 2008).

To date, there are few studies of the variability of the methodology and the requirements for precision in estimation from PRBS testing (Edwards et al., 2001, 2003; Koschate et al., 2016). In support of recent findings (Beltrame and Hughson, 2017a,b; Beltrame et al., 2017a), the purpose of this study was to describe in detail the computation of the mean normalized gain (*MNG*) and to test its consistency to characterize the $\dot{V}O_{2}$ kinetics during random exercise in humans. The *MNG* will be validated against the time-domain approach, and checked for intra-subject consistency by applying multiple PRBS protocols on different days and times of the day. In addition, the *MNG* was also evaluated considering different number of repeated tests averaged together before data modeling and different filtering techniques.

The hypothesis of this study was that the $\dot{V}O_{2}$ dynamics characterized by *MNG* during random exercise would be similar to the dynamic indices obtained by time-domain analysis, even with fewer exercise repetitions. In addition, we hypothesized that *MNG* was independent of the testing day and the time of the day, demonstrating therefore that it can be used to evaluate the individual aerobic response during random exercise in humans. These results could set the stage for advancing frequency domain analyses outside the confines of the research laboratory to assess kinetics, and therefore an index of physical fitness, in activities common to daily living or athletic training.

## Materials and methods

### Study design

Eight healthy, young adults (28 ± 6 years old, 175 ± 7 cm, and 79 ± 13 kg), who were not athletically trained, participated in this study. All participants visited the laboratory four separate times to complete submaximal exercise protocols. The study was approved by the Office of Human Research of the University of Waterloo and was in agreement with Declaration of Helsinki. Participants provided written informed consent after receiving full study details and being made aware that they could withdraw at any time without penalty.

On each visit, three successive PRBS sequences were completed in a single, continuous session. The signal related to the first PRBS in each visit was excluded *a priori* as a warm-up (Hughson et al., 1990) and the remaining PRBS protocols were numbered in sequence (1–8) and considered under separate conditions defined by their time of day (morning and afternoon, separated by 6 h) and by their different days (day 1 and day 2, separated by 1 week) to test consistency of the $\dot{V}O_{2}$ dynamics characterization. The datasets were also analyzed considering different filtering methods for pre-processing including moving average, multiple tests averaging and low-pass filtering (see Table 1).

**Table 1:** Description of the pseudorandom binary sequence (PRBS) protocols (1–8) used to test the influence of some conditions and data pre-processing over the oxygen uptake dynamics.

	**Conditions**	**Factor**	**PRBS evaluated**
Consitency	Time of the day	Morning	Average of 1+2+5+6
		Afternoon	Average of 3+4+7+8
	Day	Day 1	Average of 1+2+3+4
		Day 2	Average of 5+6+7+8
Filtering	Moving average	3 s	1
		5 s	1
		7 s	1
	Repetitions without low-pass filtering	1 repetition	1
		2 repetitions	Average of 1+2
		3 repetitions	Average of 1+2+3
		4 repetitions	Average of 1+2+3+4
		5 repetitions	Average of 1+2+3+4+5
		6 repetitions	Average of 1+2+3+4+5+6
		7 repetitions	Average of 1+2+3+4+5+6+7
		8 repetitions	Average of 1+2+3+4+5+6+7+8
	Repetitions with low-pass filtering	1 repetition	1
		2 repetitions	Average of 1+2
		3 repetitions	Average of 1+2+3
		4 repetitions	Average of 1+2+3+4
		5 repetitions	Average of 1+2+3+4+5
		6 repetitions	Average of 1+2+3+4+5+6
		7 repetitions	Average of 1+2+3+4+5+6+7
		8 repetitions	Average of 1+2+3+4+5+6+7+8

### Pseudorandom binary sequence exercise test (PRBS)

**Figure 1:**
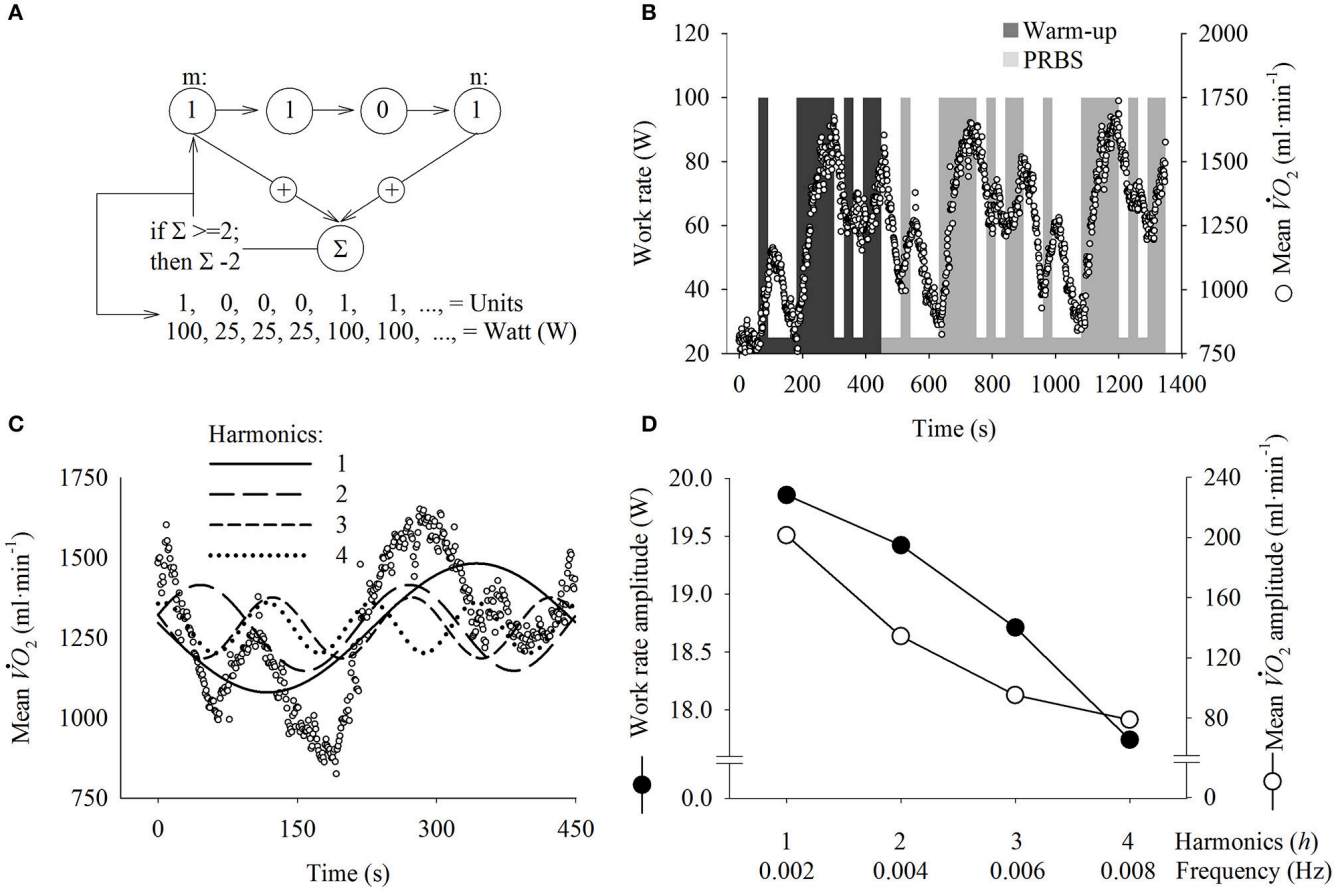
**(A)** Illustration of the 4-stage shift register used to generate the pseudorandom binary sequence protocol (PRBS). The module addition feedback (∑) sums the first and fourth stage values and tests the “if” statement. The value is then inserted into the first stage and the entire system shifts to the right, and the sequence repeats after 15 values. Each unit is maintained for 30s to create the PRBS protocol in the time-domain **(B)**. The system input stimulates the oxygen uptake ($\dot{V}O_{2}$) response (−), here represented by the mean signal of all participants (*n* = 8) during the first visit. The $\dot{V}O_{2}$ data regarding the first PRBS sequence (warm-up) were excluded *a priori*. **(C)** To illustrate the frequency domain analysis, Fourier transformations were used to decompose the $\dot{V}O_{2}$ time-domain response of the second PRBS sequence into amplitudes of sinusoidal functions at specific frequencies (i.e., harmonics). The amplitudes of the system input (i.e., work rate in Watts) and output (i.e., $\dot{V}O_{2}$) at different frequencies are displayed in **(D)**.

All exercise tests were performed on an electrically braked cycle ergometer controlled by an external, pre-programmed module (Lode Excalibur Sport, Lode B.V., Groningen, Netherlands). The PRBS protocol (Figure 1B) was generated by a digital shift register with an adder module feedback (Bennett et al., 1981; Hughson et al., 1990; Beltrame and Hughson, 2017b) (Figure 1A). The target *Ẇ* (reached after <1.5 s of transition following a modification of the ergometer controller) was 25 or 100 W, and the cadence was maintained at ≈ 1 Hz. As described in Figure 1, the PRBS protocol comprised 15 units (25 or 100 W) for 30 s (total of 450 s for each PRBS). According to previous studies (Beltrame and Hughson, 2017a,b), the highest *Ẇ* used in the current study (i.e., 100 W) is constrained to moderate intensity exercise thus avoiding the presence of system distortions that might influence the system temporal analysis.

### Data acquisition and analysis

The $\dot{V}O_{2}$ data were measured breath-by-breath by the V_max_ system (CareFusion, San Diego, CA, USA) that estimates the air volume through a low resistance mass flow sensor (accuracy of >97%), the O_2_ pressure by an electro-chemical cell (accuracy of >99%), and the *CO*_2_ pressure by an infrared light with a thermopile (accuracy of >99%). The gas concentrations and air volume/flow were calibrated following manufacturer's specifications before each test. The raw breath-by-breath $\dot{V}O_{2}$ data were linearly interpolated second-by-second by the *interpolation transform* in SigmaPlot 12.5 software (Systat Software, San Jose, CA, USA)

When appropriate, different filtering techniques were applied over $\dot{V}O_{2}$ data. The moving average filtering level varied between different window sizes (3, 5, or 7 s) and the low-pass filter considered a cutoff frequency of 0.075 Hz following previous literature (Harper et al., 2006; Schlup et al., 2015). Filters were implemented in Origin 9.1 software (OriginLab Corp. Northampton, MA, USA). Afterwards, the $\dot{V}O_{2}$ data were time aligned and ensemble-averaged to obtain a single response per participant from different combinations of repetitions as described in Table 1.

### Frequency domain analysis

The *MNG* was calculated based on the frequency-domain data transformation (Hughson et al., 1990). The datasets used for *MNG* calculations are described in Table 1; however, the last condition (low-pass filtering) was not tested due to the embedded filtering characteristics of the *MNG* estimation that has a cut-off frequency lower than 0.075 Hz (explained below).

The first step in the calculation of MNG required frequency domain analysis at each of the first four harmonics. Data from the exercise input (*Ẇ*) and output ($\dot{V}O_{2}$) were analyzed using a standard Discrete Fourier Transformation algorithm (Smith, 1999). The following sinusoidal function was solved for harmonics 1–4 as described previously (Hughson et al., 1990):

(1) $$\begin{array}{l} \dot{V}O_{2\left(t\right)\;}=\;a_{0}+\;2\;*\;\sum_{h=1}^{4}(A_{h}\;*\;\cos\left(2\pi \;*\;h\;*\;f_{1}\;*\;t\right)\\ \;+\;B_{h}\;*\;\sin\;\left(2\pi \;*\;h\;*\;f_{1}\;*\;t\right)) \end{array}$$

where *t* is the time of the PRBS, *a*_0_ is average response during the entire PRBS, *f*_1_ is the fundamental frequency calculated as the inverse of the protocol length of 450 s (i.e., 1/450 or 0.0022 Hz). As depicted in Figures 1C,D, the *f*_1_ can be defined as the lowest frequency evaluated and the subsequent frequencies were defined by the product between *f*_1_ and the harmonics (*h*). Harmonics are integer numbers that define how many complete sinusoidal cycles into which the time series signal was decomposed. The *A*_*h*_ and *B*_*h*_ are the cosine and sine amplitudes for a given harmonic *h*, respectively. From *A*_*h*_ and *B*_*h*_, the sinusoidal amplitude (*Amp*) was computed for each *h* (Figure 1D) by:

(2) $$Amp_{n\;}=\sqrt{A_{h}^{2}+B_{h}^{2}}$$

The system gain was calculated at each *h* (*gAmp*_*h*_) from the relationship for the individual input:output relationships at that harmonic by the ratio:

(3) $$gAmp_{h}\;=\;\dot{V}O_{2}Amp_{h}/\dot{W}Amp_{h}$$

### Isolating temporal dynamics from the frequency domain responses

**Figure 2:**
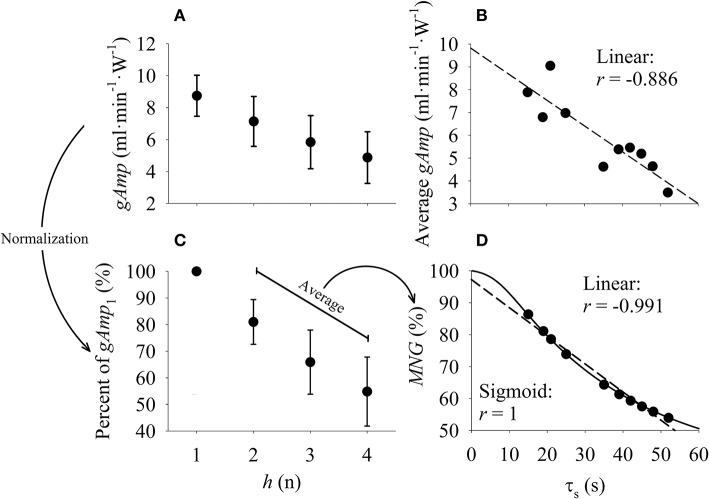
**(A)** Mean ± SD of 10 simulations of the system gain (*gAmp*) calculated in the frequency domain from data generated using 10 different values of system time constants (τ_*s*_), amplitudes and baselines (see Table 2). **(B)** linear (– –) relationship between τ_*s*_ and the average absolute oxygen uptake gain at each tested harmonic (*h*) for each of the 10 simulations. **(C)** mean ± SD of data displayed in A normalized by the *gAmp* at *h*_1_. The mean normalized gain (*MNG*) was calculated as the mean of the *gAmp* between *h*_2_, *h*_3_, and *h*_4_ (please see text and Equation 4). **(D)** relationship between τ_*s*_ and *MNG*. This relationship was fitted by a linear (– –) and sigmoid (–) function. Notice in **(D)** that the normalization procedure isolated the relationship between amplitude and τ_*s*_ from other sources of system distortion such as system gain and baseline. The correlation coefficient “*r”* was used to indicate the degree of correlation between τ_*s*_ and *MNG*. Please see text for further details regarding the sigmoid function.

The *MNG* was calculated based on the normalization of individual harmonic gains. As illustrated by the arrow between Figures 2A,C, the system gains were normalized as a percentage of the *gAmp* at *h*_1_ (i.e., *gAmp*_1_) (Hoffmann et al., 1994; Beltrame and Hughson, 2017a,b). This normalization isolated the temporal dynamics of the system by removing the influences of the total gain (i.e., steady-state gain) across the harmonic amplitudes (Hoffmann et al., 1992). Therefore, based on a previous concept (Shmilovitz, 2005; Beltrame et al., 2017a; Beltrame and Hughson, 2017a,b), the new index of system dynamics called mean normalized system gain (*MNG*, expressed in % in Figure 2D) was obtained by the average of the normalized system gains (smaller arrow in Figure 2C) of the harmonics 2, 3, and 4 (*h* = 2, 3, and 4) following the equation:

(4) $$MNG\;=\;(\sum_{2}^{4}gAmp_{h}/3\;*\;100)/gAmp_{1}.$$

### Time-domain analysis

The time-domain analysis of the $\dot{V}O_{2}$ data was conducted on a segment of the PRBS for comparison to the *MNG* obtained by frequency domain analysis. The data window length for time-domain analysis included the final 10s of a 90s period of 25 W followed by 120s at 100 W (starting at the 180th second of the PRBS protocol). This exercise window corresponded to the longest period without input variation, thus the best window for time-domain analysis within the PRBS protocol. The following equation was used to fit the $\dot{V}O_{2}$ data (Hughson and Morrissey, 1982; Whipp et al., 1982):

(5) $$\dot{V}O_{2(t)}\;=\;a_{0}+a\left(1-e^{-(t-TD)/\tau }\right)$$

where *t* is time; *a*_0_ is the baseline at 25 W; *a* is the steady state amplitude at 100 W; τ is time constant (i.e., the “speed” of the system) and *TD* is the time delay of the exponential function onset. The initial data associated with the cardio-dynamic component (20 s) were excluded before data fitting. The mean response time (*MRT*) was calculated by adding t and *TD* (Macdonald et al., 1997). The quality of the fitting was assured by the analysis of squared error, coefficient of determination (*r*^2^), 95% confidence interval band (*CI*_95_) of the model (Fawkner et al., 2002; Keir et al., 2016) and the significance level (*p*-value) of the estimated parameters. The comparison between *MNG* and τ, both derived from measured $\dot{V}O_{2}$ data, has the purpose to experimentally support the expected correlation between both parameters (further demonstrated by computer simulations). It is worth to mention that this study was not designed to obtain τ under ideal condition since the dataset used for this purpose was, and must be for a fairer comparison between indexes, nested within the PRBS protocol. Therefore, the time domain approach was only used as a supplementary analysis to validate this new index (i.e., *MNG*) estimated from frequency domain response.

### *In silico* simulations: *MNG* vs. time constant

Simulations of the $\dot{V}O_{2}$ response stimulated by PRBS input were performed to determine the relationship between the time constant (for the simulations denoted τ_*s*_) and *MNG*, derived from time- and frequency-domain analysis, respectively. Similarly to τ_*s*_, *MNG* should extract information regarding the $\dot{V}O_{2}$ system adaptation speed from random exercise stimulus which ultimately is associated with aerobic fitness (Hagberg et al., 1980; Powers et al., 1985; Chilibeck et al., 1995; Hughson, 2009).

As previously described elsewhere (Hoffmann et al., 2013; Beltrame and Hughson, 2017a), an algorithm was created to simulate the $\dot{V}O_{2}$ response to PRBS considering the function described above in the *Time-domain analysis* section. This algorithm assumed a linear static and dynamic $\dot{V}O_{2}$ gain (Eßfeld et al., 1991; Hoffmann et al., 1992) with no time delay, as expected in muscular $\dot{V}O_{2}$ response (Hoffmann et al., 2013). Firstly, ten simulations were generated by arbitrarily selecting different combinations between *a*_0_, *a*_1_ and τ_*s*_ as described in Table 2. The average *Amp* gain between the analyzed frequencies and the *MNG* are also described in Table 2. For further discussion, the physiological range of τ and τ_*s*_ was defined as 10 < τ and τ_*s*_ < 100 s.

**Table 2:** Description of the parameter used for the computer simulations and the parameters obtained by frequency-domain analysis.

**Simulation**	**1**	**2**	**3**	**4**	**5**	**6**	**7**	**8**	**9**	**10**
*a*_0_ (ml·min^−1^)	300	400	350	250	200	150	125	350	250	330
*a*_1_ (ml˙min^−1^)	700	800	750	900	750	600	800	600	750	650
τ_*s*_ (s)	15	45	25	21	39	52	42	35	48	19
Average *gAmp* (ml.min^−1^*Ẇ*^−1^)	7.9	5.2	7.0	9.0	5.4	3.5	5.5	4.6	4.6	6.8
*MNG* (%)	86	58	74	79	61	54	59	64	56	81

*a_0_, baseline; a_1_, steady state amplitude; τ_s_, exponential time constant of the simulations; gAmp, gain amplitude; MNG, mean normalized gain amplitude*.

**Figure 3:**
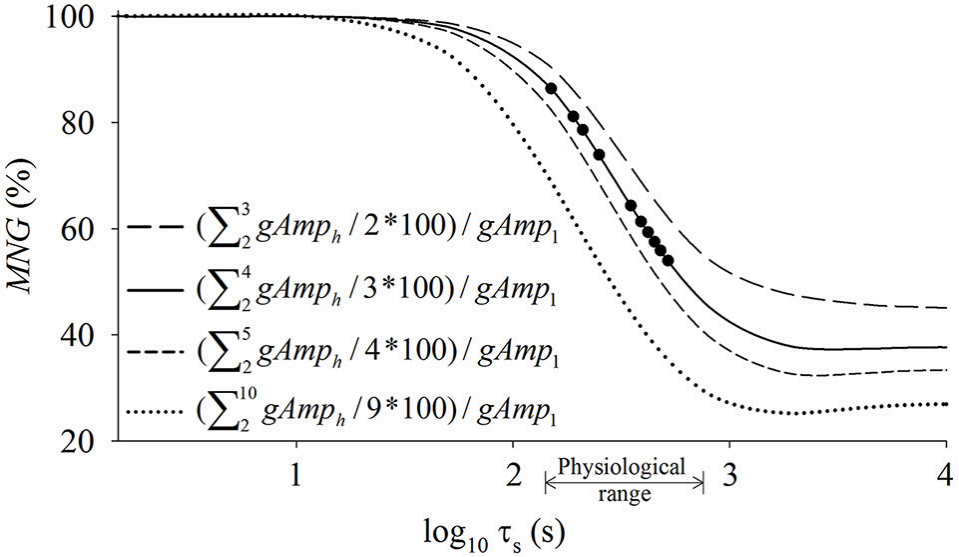
Computer simulations were performed to generate different oxygen uptake ($\dot{V}O_{2}$) responses considering different values of time constant (τ_*s*_) that defines the speed of the $\dot{V}O_{2}$ adjustment to random exercise. The $\dot{V}O_{2}$ data were transformed to frequency domain and the mean normalized gain amplitude (*MNG*) was obtained considering the normalized system gain obtained from different frequency ranges. The equations describe how the *MNG* was obtained from the $\dot{V}O_{2}$ data. The x-axis scale was converted to log_10_ for a better visualization of the sigmoidal characteristics of the relationship between *MNG* and τ_*s*_. The symbols “•” represent the simulated data from Figure 2D. Please see Equation (4) and text for further details regarding the equation parameters and procedures. Notice that the linear portion of the sigmoid function is always located at the physiological portion of tau values (i.e., from 10 to 100 s).

The system gains (Figure 2A) are dependent in the simulations on the values of *a*_0_ and *a*_1_ (amplitude components) as well as τ_*s*_ (speed component). Since the extraction of the system temporal characteristics (such as τ_*s*_) is the goal of our index, *a*_0_ and *a*_1_ can be considered as confounding factors. Thus, as shown in Figure 2B, the simple average of the absolute gains across the tested frequencies was not able to perfectly predict τ_*s*_; however, the normalization method used to obtain the *MNG* was able to better isolate τ_*s*_ from the different system gains and baselines (Figure 2D). In addition to the simulations that used a physiological range of the parameters, more simulations were performed to further investigate the expected behavior of the relationship between *MNG* and τ_*s*_. These simulations (*n* = 10) used a constant *a*_0_ and *a*_1_ but varied τ_*s*_ to extreme values (0.001, 0.1, 1, 5, 15, 35, 80, 200, 500, and 1500 s). Different combinations of harmonics (i.e., *h*′s) to calculate *MNG* (derived from equation 4) were also tested. Specifically, the following combinations between *h* were tested for the *MNG* calculation: 2 ≤ *h* ≤ 3, 2 ≤ *h* ≤ 4, 2 ≤ *h* ≤ 5, and 2 ≤ *h* ≤ 10. The relationship between *MNG* and τ_*s*_ (Figures 2D, 3) was described by a sigmoid function. The x-axis scale in Figure 3 was converted to log_10_ for a better visualization of this relationship.

If more normalized gains from the simulated linear systems are considered into *MNG* calculation (equations in Figure 3), the sigmoid is shifted to left and the plateau for longer τ_*s*_ became smaller. However, the physiological range (arrow in Figure 3) of τ_*s*_ was always located at the approximately-linear portion of the sigmoid, independently of the number of harmonics used to calculate *MNG*. The improvements (measured by the *r*-value) from the sigmoidal to the linear fitting was minimal (or 0.9% as displayed in Figure 2D). Therefore, considering the model degree of freedom, the physiological range in τ, and the error associated with the τ estimation from real data, the relationship between *MNG* and τ was simplified to a linear relationship (– –, in Figures 2B,D). The system analysis of the current study was limited to the fourth harmonic (Figure 2A, *h* = 4 or 0.008 Hz) because the ${\dot{V}O}_{2}$ data, and presumably the aerobic system response, can be analyzed as a first order linear system (Hoffmann et al., 1992). It is important to adhere to the linearity principle to avoid misinterpretation about the ${\dot{V}O}_{2}$ dynamics that might not be driven directly by work rate effect on the metabolic response but by circulatory distortions at frequencies higher than ≈0.01 Hz (Hoffmann et al., 1992). Kinetics analyses and data simulations were performed by a certified (#100-314-4110) LabVIEW associated developer (National Instruments, Austin, TX, USA).

### Statistical analysis

According to Shapiro-Wilk test, most of the data were normally distributed. The *MNG*, τ and *MRT* were compared between different conditions (time of day, or different days) by paired *t*-test. One way repeated measures ANOVA was used to test the impact of the moving average filtering level on *MNG* by comparing different average window sizes (3, 5, or 7 s) applied over the first PRBS protocol with the signal from this same protocol without filter. The *MNG* was compared between different exercise repetitions (1–8) by one way repeated measures ANOVA. Statistical differences in τ and *MRT* obtained from different exercise repetitions (1–8) with or without low-pass filtering were assessed by two way repeated measures ANOVA. Student-Newman-Keuls method was selected for *post*-*hoc* analysis. When appropriate, sample size was calculated using Student *t*-test or paired *t*-test as reference test and considering the SD of the *MNG* or τ, both estimated from eight exercise repetitions without filtering, with the power set at 0.8. The linear correlation was measured by Pearson product-moment correlation coefficient (*r*) and coefficient of determination (*r*^2^). The agreement level was assessed by Bland-Altman plot and *CI*_95_ (Altman and Bland, 1983). The *CI*_95_ of τ and *MNG* was obtained as 1.96^*^SD of the group response and, for the sake of comparison between these parameters, the *CI*_95_ was reported as percentage of the group mean. For all statistical tests, the statistical significance (*p*) was set at < 0.05. Statistical analysis was conducted in SigmaPlot 12.5 software (Systat Software, San Jose, CA, USA).

## Results

### *MNG* vs. τ

The parameters from time-domain analysis (*a*_0_, *a*_1_, τ, and *TD*) and the *MNG* obtained by frequency-domain analysis based on different exercise repetition combinations and frequency filtering levels are reported in Table 3. The parameter estimates of τ and *MRT* were not statistically (*p* > 0.05) different between different exercise repetitions, nor were they different with or without low-pass filtering.

**Table 3:** Parameter obtained by time-domain oxygen uptake analysis considering different combinations of exercise repetitions (1–8) with our without low-pass filtering (0.075 Hz).

**Repetitions**	**1**	**2**	**3**	**4**	**5**	**6**	**7**	**8**
**WITHOUT LOW-PASS FILTERING**								
*a*_0_	888 ± 132	892 ± 121	903 ± 143	924 ± 141	932 ± 134	941 ± 135	944 ± 130	955 ± 123
*a*_1_	790 ± 215	811 ± 176	766 ± 98	759 ± 77	737 ± 69	740 ± 73	744 ± 63	733 ± 71
τ	34.1 ± 27.9	39.7 ± 18.9	34.5 ± 13.3	33.3 ± 10.9	31.2 ± 9.4	33.3 ± 10.7	34.5 ± 9.4	34.1 ± 10.2
*TD*	18.1 ± 12.4	14.7 ± 6.8	15.2 ± 4.5	15.2 ± 4.2	16.0 ± 4.1	15.8 ± 4.0	15.4 ± 4.1	15.8 ± 4.4
*MRT*	52.2 ± 20.1	54.4 ± 15.6	49.7 ± 13.4	48.5 ± 10.9	47.2 ± 9.7	49.1 ± 11.7	50.0 ± 10.5	50.0 ± 11.0
*MNG*	60 ± 9	57 ± 7	57 ± 7	56 ± 7	56 ± 7	56 ± 7	57 ± 7	58 ± 7
**WITH LOW-PASS FILTERING (0.075 Hz)**								
*a*_0_	887 ± 138	902 ± 119	904 ± 147	924 ± 144	932 ± 136	940 ± 137	942 ± 132	955 ± 125
*a*_1_	797 ± 22	763 ± 12	768 ± 100	761 ± 79	740 ± 72	744 ± 75	748 ± 64	735 ± 72
τ	35.1 ± 29.4	33.2 ± 15.8	35.2 ± 13.8	33.8 ± 10.9	31.7 ± 9.50	33.7 ± 10.7	35.0 ± 9.35	34.5 ± 10.1
*TD*	17.7 ± 11.8	17.2 ± 8.4	14.9 ± 4.4	14.9 ± 4.0	15.8 ± 4.0	15.5 ± 3.9	15.1 ± 3.9	15.6 ± 4.2
*MRT*	52.8 ± 21.6	50.5 ± 12.7	50.2 ± 13.7	48.8 ± 11.1	47.6 ± 9.90	49.3 ± 11.9	50.2 ± 10.7	50.2 ± 11.1

*The mean normalized gain (MNG) is also described for different exercise repetitions. a_0_, baseline; a_1_, steady state amplitude; τ, exponential time constant; TD, time delay of the exponential function; MRT, mean response time*.

**Figure 4:**
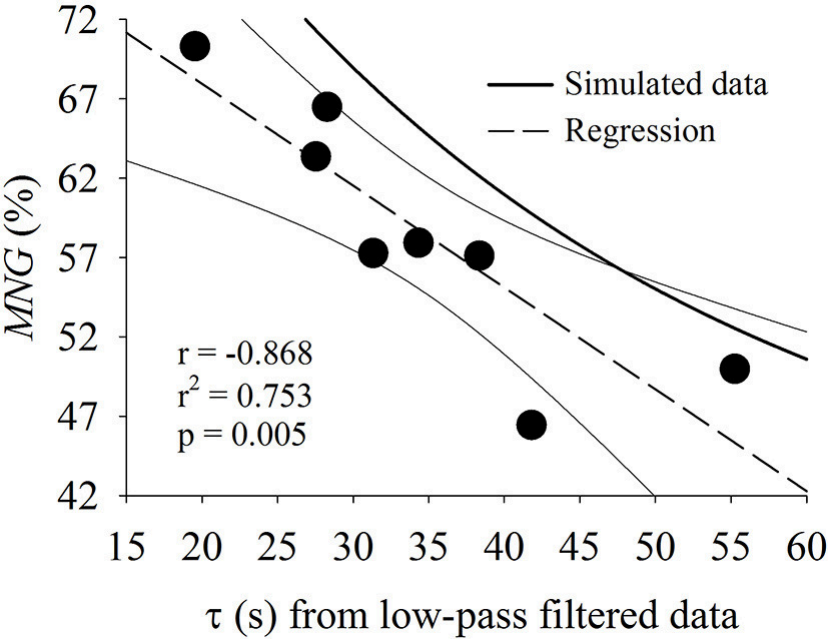
Relationship between time constant (τ) calculated from low-pass filtered oxygen uptake data and mean normalized gain (*MNG*). Both indexes were obtained from eight repetitions for all participants (*n* = 8) of the pseudorandom binary sequence test. The individual values (black circles), the regression line (dashed) and 95% CI (solid thin lines) are shown in comparison to the simulated data (solid thick line for *MNG* vs. τ_*s*_ as in Figures 2C).

Figure 4 shows the correlation between τ and *MNG* obtained by time- and frequency-domain analysis, respectively. Both parameters were obtained based on eight-repetition dataset and τ was also obtained from low-pass filtered data. For visual comparison, the simulated data displayed in Figure 2D are also plotted in Figure 4. The *MNG* of the experimental data were consistently less for all participants than the values obtained with simulated data for any value of τ.

### Influence of time of day and between days

**Figure 5:**
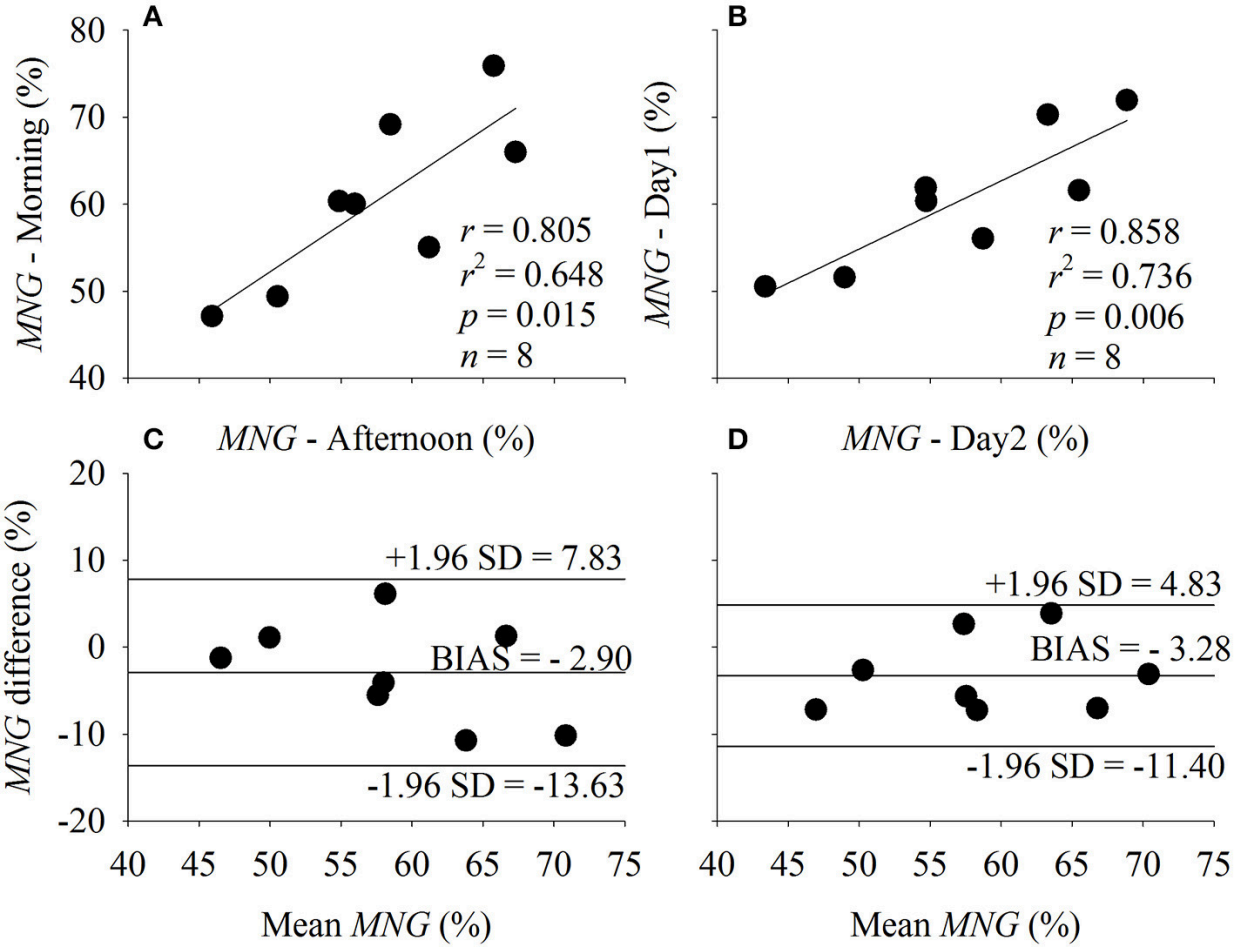
Correlation between the mean normalized gain amplitude (*MNG*) estimated under the influence of different time of the day (**A**, Morning vs. Afternoon) and different day of testing (**B**, Day 1 vs. Day 2). The agreement level between the factors plotted in **(A,B)** are displayed in **(C,D)**, respectively. *r*, Pearson's correlation level; *p*, statistical significance level; and *n*, sample size.

**Figure 6:**
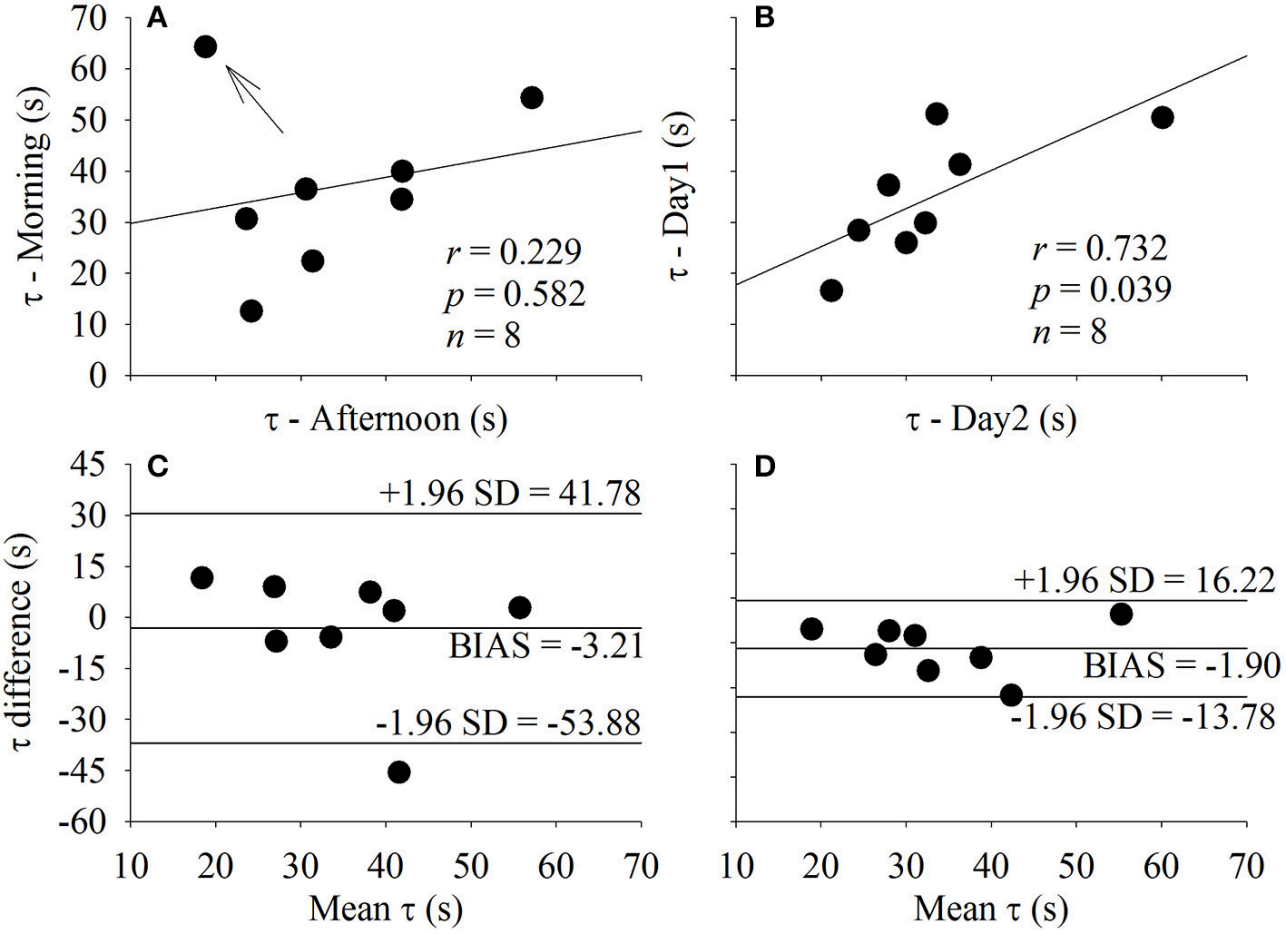
Correlation between the time constant (τ) estimated under the influence of different time of the day (**A**, Morning vs. Afternoon) and different day of testing (**B**, Day 1 vs. Day 2). The agreement level between the factors plotted in **(A,B)** are displayed in **(C,D)**, respectively. The arrow indicates an outlier. *r*, Pearson's correlation level; *p*, statistical significance level; *n*, sample size.

The τ, *MRT*, and *MNG* were not statistically different when compared across time of day (*p* > 0.05) and between days (*p* > 0.05). The relationship and the agreement level of the *MNG* (Figure 5) and τ (Figure 6) obtained during the morning and afternoon (Figures 6A,C) and in different days (Figures 6B,D). The *MNG* was strongly correlated between the time of day (morning vs. afternoon) and between days (day 1 vs. day 2). The bias of the *MNG* calculation represented 4.92 and 5.57% of the total *MNG* variation during the different time of the day and between days, respectively. The *CI*_95_ were equivalent to 18.21 and 13.78% of the total *MNG* variation of the sample for the different time of the day and between days, respectively. The τ was not correlated between the time of day (morning vs. afternoon) possibly due to the outlier identified in Figure 6A by the arrow. However, τ was strongly correlated between days (day 1 vs. day 2). The bias of the τ calculation represented 9.11 and 5.58% of the total τ variation during the different time of the day and between days, respectively. The *CI*_95_ were equivalent to 95.28 and 47.28% of the total τ variation of the sample for the different time of the day and between days, respectively.

### Influence of the averaging level and number of repetitions

The different moving average filtering levels (3, 5, or 7 s) have not impacted the *MNG* estimation during the first PRBS protocol. The correlation coefficient *r* was 0.99 for all levels in comparison to the signal without moving average filtering. In addition, the bias and the *CI*_95_ between all filtering levels and the signal without filtering was minimal (< ≈1%).

**Figure 7:**
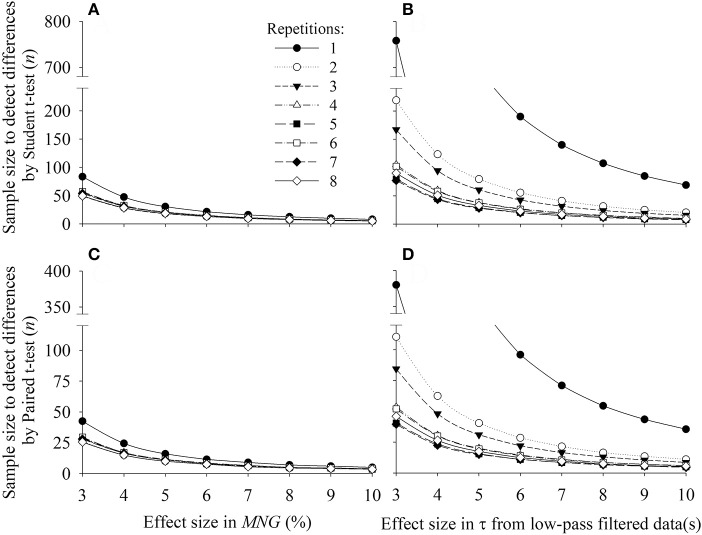
Relationship between effect size of the mean normalized gain amplitude (*MNG*) or time constant from low-pass filtered data (τ) with the sample size needed to find statistical significance by student *t*-test **(A,B)** or paired *t*-test **(C,D)** considering different number of exercise repetitions (symbols). The desired power and the significance level considered for the sample size calculations were 0.8 and 0.05, respectively.

Figure 7 illustrates the sample size needed to find statistical significance for a given effect size (changes in *MNG* or τ) by Student *t*-test (Figures 7A,B for *MNG* and τ, respectively) or Paired *t*-test (Figures 7C,D for *MNG* and τ, respectively) considering different number of repetitions averaged together before data analysis (time or frequency-domain analysis). The relationship between sample size and the effect size suggested an exponential-decay-like function. The sample size of τ was more dependent on the number of repetitions in comparison to *MNG* in both tests (Student and Paired *t*-test).

**Figure 8:**
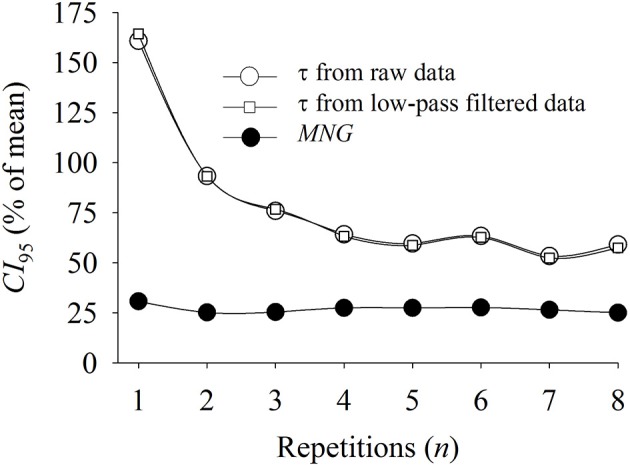
Relationship between the confidence interval (*CI*_95_, reported as percentage of the mean) of the time constant (τ) and mean normalized gain (*MNG*) obtained from different numbers of exercice repetitions. The τ was obtained from raw and filtered (0.075 Hz) oxygen uptake data. The τ *CI*_95_ stabilized at ≈70% after four exercise repetitions while the *MNG CI*_95_ was constant at ≈30% across all exercise repetitions.

The repeated measures ANOVA showed that the *MNG*, as well as τ, were not statistically different (*p* > 0.05) between different exercise repetitions. However, as depicted in Figure 8, the *CI*_95_ normalized by the group mean response of τ and *MNG* differed when different numbers of PRBS were ensemble-averaged before data analysis. As expected considering the short data window and model degree of freedom, the τ *CI*_95_ was extremely high when only one exercise repetition was considered for data modeling. After that, the τ *CI*_95_ stabilized after four exercise repetitions at ≈70% for both, with or without low-pass filter. If we consider that the aerobic system temporal dynamics were unchanged across different exercise repetitions, the observed τ *CI*_95_ of 70% in filtered data can be interpreted as an intrinsic variability originating from time-domain data modeling and from variable aerobic fitness of the participants. On the other hand, the *MNG CI*_95_ seemed to be independent of the number of exercise repetitions and it was stable at ≈30% which indicated a lower method-originated variability, isolating variations of aerobic fitness from the calculation distortions. Therefore, the actual variability of the aerobic system temporal dynamics, understood as aerobic fitness, seems to be approximately 40% around the mean.

## Discussion

In agreement with our initial hypothesis, the calculation of *MNG* was able to characterize the temporal dynamics of $\dot{V}O_{2}$ to random exercise input being strongly correlated with the time-domain indicator, τ, obtained in the same persons. The *MNG* eliminated the expected differences in static gain between individuals by expressing the dynamic response as a percentage of the fundamental harmonic value. The comparison of *MNG* against the time-domain $\dot{V}O_{2}$ kinetics analysis was shown to be independent of the period of the day, the day of the test, the filtering technique used and the number of exercise repetitions ensemble-averaged before data analysis. The detection of differences in *MNG* was independent of the number of exercise repetitions for differences higher than ≈8% which correspond to a τ variation of ≈15 s. Further, these data are important for the experimental design of further studies by informing the number of repetitions necessary according to an expected effect size (Figure 7). In addition, as reported in Figure 8, *MNG* seemed to be, in comparison with τ, ≈50% less susceptible to noise than time-domain analysis thus isolating better the temporal dynamics of the aerobic response to changes in energy demand.

The breath-by-breath fluctuation (Lamarra et al., 1987) during exercise transitions adds uncertainty to time-domain parameter prediction, mainly from a single test dataset. The confidence interval of the estimated τ, and therefore the “sensitivity” to identify aerobic fitness differences, depends on the $\dot{V}O_{2}$ signal-to-noise ratio (Lamarra et al., 1987; Keir et al., 2014b), the model complexity (i.e., the degree of freedom) (Motulsky and Ransnas, 1987) and the selected data window (Bell et al., 2001; Murias et al., 2011). To increase signal-to-noise ratio, studies commonly repeat similar tests multiple times within the same session (Ozyener et al., 2001; Christensen et al., 2016) or on different days (Whipp et al., 1982; Keir et al., 2014a) and average repetition-like transitions before time-domain data modeling.

The frequency-domain analysis has some advantages over the time-domain approach. Firstly, no explicit data modeling with a degree of arbitrariness is necessary (Eßfeld et al., 1987) since the $\dot{V}O_{2}$ time series can be decomposed, and therefore rebuilt from the infinite sum of its harmonic components (Hughson et al., 1990). Second, the random noise associated with $\dot{V}O_{2}$ measured at the mouth (Lamarra et al., 1987) is filtered when transferred into frequency space, diminishing the impact of the inter-breath oscillations over the $\dot{V}O_{2}$ dynamics characterization.

The early studies from Eßfeld et al. (1991) and Hoffmann et al. (1992) were the first to normalize the system gain amplitudes by the amplitude at the fundamental harmonic (i.e., *gAmp*_1_ in Equation 4). They successfully showed that a faster $\dot{V}O_{2}$ kinetics maintained a higher normalized gain across the frequency spectrum. However, since the focus of their experiments was to investigate possible aerobic system controllers, no further comparisons were carried out to explore the applicability of this normalization for the $\dot{V}O_{2}$ temporal dynamics assessment. Other studies used the absolute system gain to infer about $\dot{V}O_{2}$ dynamics (Eßfeld et al., 1987; Hughson et al., 1990, 1991). In fact, the use of absolute gains may be sufficient for intra-subject comparisons since the system static gain seems to remain constant as the aerobic system “speeds up” after training (Christensen et al., 2016). However, for the comparison between subjects by an absolute index such as τ, the gain must be normalized.

We demonstrated by computer simulations (Table 2, Figure 2) that *MNG* was able to characterize the temporal characteristics of the aerobic system by comparing *MNG* with τ_*s*_. The *MNG* refined the ability of the Fourier transformation to separate the system dynamic gain from the static gain, isolating therefore the rate at which the aerobic system supplies the energy demand (i.e., power) from the capacity of the aerobic system to supply the demand at steady state. The latter is susceptible to inter-individual variability which confounds the interpretation of the temporal dynamics based on the system absolute gains (as demonstrated in Figure 2B).

In the experimental data (Figure 4), we demonstrated that the *MNG* was significantly correlated to τ (used as reference). The τ calculated from eight repetitions and low-pass filter still has an intrinsic non-physiological variability that could be associated with the low signal-to-noise ratio as a consequence of noise, short data window and/or elevated modeling degrees of freedom. The *CI*_95_ of the τ estimated from eight repetitions and low-pass filtered (0.075 Hz) data between all participants (*n* = 8) was 19.8 s which represented 57% of the average τ-value. In contrast, the *MNG* presented a lower variability in comparison to τ possibly due to the inherent noise reduction and the lower degrees of freedom of the proposed method. Consequently, only 75% of the *MNG* variation could explain the variations in τ, both calculated based on eight exercise repetitions (Figure 4). However, we demonstrated (Figure 8) that ≈30% of the τ variability (from total of ≈60%) seems to be a consequence of data modeling by comparing *MNG* and *CI*_95_. This might be occurring because τ, as an explicit parameter, has an intrinsic degree of freedom originating from $\dot{V}O_{2}$ data modeling (Motulsky and Ransnas, 1987). Therefore, based on Figure 8, the *MNG* seemed to “isolate” aerobic system temporal dynamics from noise better than τ obtained from $\dot{V}O_{2}$ time-domain analysis, even after eight repetitions and filtering. In addition, in agreement with previous research (Keir et al., 2014b), the *CI*_95_ of τ was more dependent on the number of exercise repetitions in comparison to *MNG* where the *CI*_95_ was independent of exercise repetitions.

The biological significance of τ and *MNG* was also demonstrated in Figures 5, 6 by estimating these indexes at different conditions described in Table 1. The temporal dynamics of the $\dot{V}O_{2}$ response, and presumably the aerobic fitness, should not suffer major changes between periods of day or the day of testing. However, *MNG* (Figure 5) seems to have lower variability associated to the method of choice in comparison to τ (Figure 6) which is in accordance to Figures 7, 8. In addition, the participant identified as an outlier (arrow in Figure 6A) was not apart from the group response in Figure 5A (*MNG*). Therefore, the unexpected behavior of the $\dot{V}O_{2}$ dynamics during the exercise transition used for τ estimation was not “transferred” to *MNG* because the source of this distortion was not periodic.

**Figure 9:**
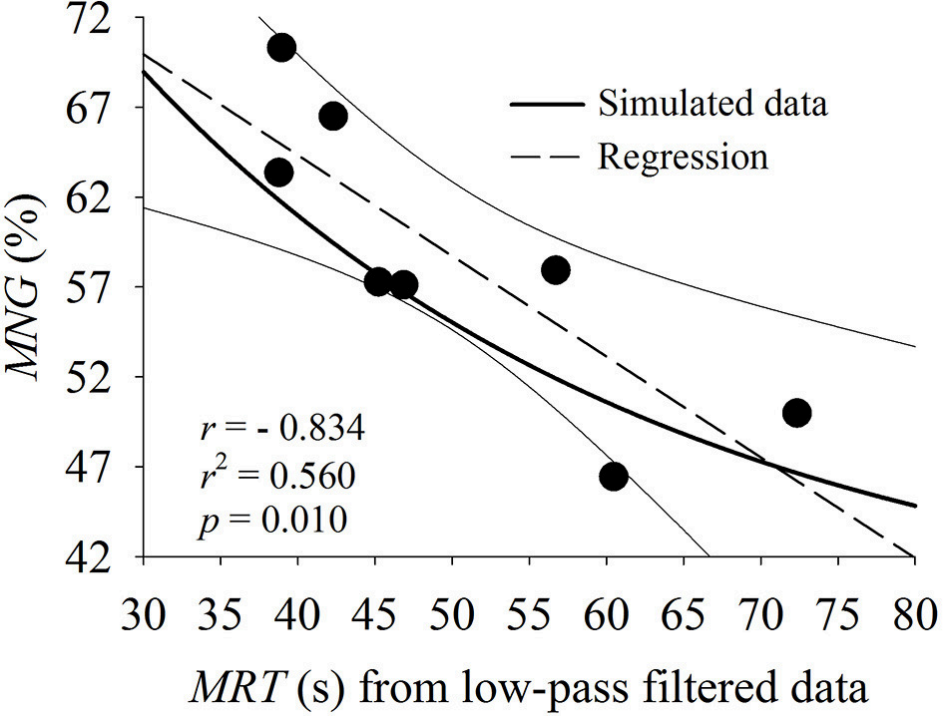
Relationship between mean response time (*MRT*) calculated from filtered oxygen uptake data and mean normalized gain (*MNG*). Both indexes were obtained from eight repetitions for all participants (*n* = 8) of the pseudorandom binary sequence test. As the individual values (black circles), the regression line (dashed) and 95% CI (solid thin lines) are shown in comparison to the simulated data (solid thick line for *MNG* vs. τ_*s*_ as in Figure 2C).

As demonstrated in Figure 4, the relationship between τ and *MNG* was systematically below the simulated data (*MNG* vs. τ_*s*_, Figures 4, 2C). There are two possible explanations for this. Firstly, following Hoffmann et al. (2013) and as expected to occur in the muscle, the simulations were generated based on a non-delayed exponential response (single time constant τ_*s*_, no *TD*). However, the $\dot{V}O_{2}$ response at the mouth is classically described as a delayed exponential response (single time constant τ with a *TD*). The addition of the *TD* term to the fitting model is a mathematical way to account for the “latency” period when the muscle responses have not started to be expressed at the mouth level due to circulatory transit time. Like the phase shift obtained from frequency-domain analysis (Eßfeld et al., 1987), the parameter *TD* has an elevated variability between subjects without main physiological relevance. Therefore, comparing the exponential characteristics (i.e., τ) of the $\dot{V}O_{2}$ response at the mouth to the simulated data τ_*s*_ appears to show an incorrect gain amplitude generated at the muscle in higher frequencies effectively “slowing down” the response in frequency domain (i.e., lower *MNG*-values). A possible way to account for this issue is to consider the sum of τ and *TD*, or the mean response time (*MRT*), as the “effective” muscular $\dot{V}O_{2}$ time constant measured at the mouth level (Linnarsson, 1974; Whipp and Ward, 1990). In comparison to Figure 4 and as depicted in Figure 9, the addition of *TD* term brings the relationship between *MRT* and *MNG* in line with the simulated data. Despite the apparent differences in *r* and *p*-values for the *MNG* vs. *MRT* compared to the *MNG* vs. τ (*r* = −0.802, *p* = 0.016 and *r* = −0.855, *p* = 0.006 respectively for 8 repetitions), the *CI*_95_ and the squared error were not statistically different (*p* > 0.05 by paired *t*-test) considering the individual responses. Therefore, the inclusion of the *TD* did not alter the relationship between *MNG* and the time-domain dynamics indicators (τ or *MRT*). It is known that *TD* does not carry biological information (Eßfeld et al., 1987; Hoffmann et al., 2013) and its inclusion is commonly related to data modeling strategy (Whipp and Ward, 1990).

An alternative explanation of why the *MNG* based on the experimental data is below the simulated data (Figure 4) is based on the $\dot{V}O_{2}$ system linearity. The $\dot{V}O_{2}$ measured at the mouth presented a certain degree of energy dispersion across the spectrum due to circulatory distortions and/or all the assumptions that are necessary to obtain an estimate of $\dot{V}O_{2}$ from ventilatory and gas concentration signals. In contrast, the data simulation was based on a purely linear system that did not present any source of distortion beyond the one related to the exercise stimulus. In the simulations, all energy applied to the system was perfectly converted into the same-order output response by the superposition law, maintaining a higher gain across the frequencies. The possibility exists that even in the range of input stimulation frequencies assumed to result in linear output ($\dot{V}O_{2}$) (Hoffmann et al., 1992) that non-linearities exist effectively lowering the system response at the higher frequencies. It was previously speculated (Hughson et al., 1990; Hoffmann et al., 1992) that distortions of the circulatory system which includes *O*_2_ stores oscillations, variable muscle-to-lungs transit time and blood venous volume (Hoffmann et al., 2013) might influence the expression of the $\dot{V}O_{2}$ dynamics at the mouth level at high frequencies.

Another advantage of *MNG* over time-domain-derived temporal indexes is that it does not require an arbitrary decision for data modeling regarding the length of the cardio-dynamic phase that is variable between participants (Murias et al., 2011). The *MNG* is estimated from periods longer than 112 s thus this index does not reflect the cardio-dynamic components during exercise transitions.

## Limitations

The intrinsic degree of uncertainty associated with τ estimated from the exponential modeling precludes the use of τ as a “gold standard” method to validate the use of *MNG* to assess the system temporal dynamics. The *CI*_95_ of the relationship between τ (and *MRT*) with *MNG* might be influenced by the elevated *CI*_95_ of τ estimation (and *TD* for *MRT*). Therefore, there is an expected source of error also in the reference method (time-domain) which complicates the validation method. Our data showed that a faster $\dot{V}O_{2}$ response will be translated to a higher *MNG* or a lower τ and *MRT*; however, the ability of the *MNG* to extract this information from $\dot{V}O_{2}$ data seemed optimized and more sensitive to detect differences in the system temporal dynamics due to its inherent filtering characteristics and the lower degrees of freedom.

As a Fourier transformation criterion, the proposed method assumes a symmetrical $\dot{V}O_{2}$ dynamic between the exercise onset and recovery transitions. However, the $\dot{V}O_{2}$ signal may be composed of asymmetries between these two phases during exercise intensities higher than moderate (Ozyener et al., 2001; Markovitz et al., 2004). The highest intensity used in the current study (100 watts) was restricted to moderate intensity (Bennett et al., 1981; Eßfeld et al., 1987; Hughson et al., 1988); therefore, the *MNG* can be compared to the τ obtained from the $\dot{V}O_{2}$ response during the onset exercise transition.

Although the frequency range selected in the current study limits the $\dot{V}O_{2}$ response to a range where the system linearity is reportedly preserved (Hoffmann et al., 1992) the *MNG* might still be susceptible to system non-linearities originating from circulatory distortions or some sort of periodic noise can be present at higher frequencies. Further studies might explore the application of specific filtering techniques (Eßfeld et al., 1987; Hoffmann et al., 1992) to remove noises/responses uncorrelated to exercise in order to increase even more the precision of the proposed index to characterize the temporal dynamics of the $\dot{V}O_{2}$ response. However, consistent with the purpose of this index, we successfully showed that a faster aerobic response can be characterized by a higher *MNG* since that the majority of the evaluated harmonics were probably linear. These results should also be verified across a wider range of participants with differing levels of physical fitness and health status.

## Conclusion

Characterization of physical fitness has classically been conducted by measurement of maximal $\dot{V}O_{2}$ (Astrand and Saltin, 1961; Drake et al., 1968). Varying levels of physical fitness and the effects of training programs are also associated with differing kinetics of adaptation of $\dot{V}O_{2}$, expressed by τ, to the challenge of a step increase in *Ẇ* (Phillips et al., 1995). The temporal characteristics of the oxygen uptake ($\dot{V}O_{2}$) dynamic during moderate exercise have previously been related to maximal aerobic power (Beltrame and Hughson, 2017b) and a faster $\dot{V}O_{2}$ response was associated with a better aerobic fitness (Powers et al., 1985; Norris and Petersen, 1998), functional mobility (Alexander et al., 2003), and disease prognosis (Borghi-Silva et al., 2012). This study, beyond demonstrating how to compute, validated a new method to assess $\dot{V}O_{2}$ dynamics in random exercises more typical of daily life.

The *MNG* can be used to identify changes in the temporal aerobic system dynamics. The applicability of our findings may extend beyond controlled exercise protocols as shown with simulated activities of daily living (Beltrame et al., 2017a) and in freely moving daily life (Beltrame et al., 2017b). Indeed, *MNG* has the potential importance to rehabilitation programs, exercise prescription and fitness evaluation where the temporal dynamics of the aerobic response might be related to aerobic power (Beltrame and Hughson, 2017b). The inherent filtering characteristics, the need for no model assumption and the low variability between days and time of the day seems to make *MNG* attractive for the evaluation of the aerobic system temporal dynamics. Additionally, because *MNG* is expressed as a percent of the fundamental harmonic, it can be applied to comparisons of system dynamics across the variables contributing to the delivery and utilization of oxygen independent of their formal units. In conclusion, the present study successfully validated the use of the *MNG* as a tool for aerobic system analysis based on random exercise stimulus.

## Author contributions

TB and RH conceived and designed research, performed experiments, analyzed data, interpreted results of experiments, prepared figures, drafted manuscript, edited and revised manuscript, and approved final version of manuscript.

### Conflict of interest statement

The authors declare that the research was conducted in the absence of any commercial or financial relationships that could be construed as a potential conflict of interest.

## Abbreviations

*a*_0_: Baseline
*a*_1_: Steady state amplitude
*f*_1_: Fundamental frequency
*gAmp*: System gain
*h*: Harmonic number
*MNG*: Mean normalized gain
*MRT*: Mean response time
PRBS: Pseudorandom binary sequence
τ: Time constant
*TD*: Time delay of the exponential function
τ_*s*_: Time constant of simulations
$\dot{V}O_{2}$: Oxygen uptake
*Ẇ*: Work rate.
